# Time and Memory Efficient Online Piecewise Linear Approximation of Sensor Signals

**DOI:** 10.3390/s18061672

**Published:** 2018-05-23

**Authors:** Florian Grützmacher, Benjamin Beichler, Albert Hein, Thomas Kirste, Christian Haubelt

**Affiliations:** 1 Institute of Applied Microelectronics and Computer Engineering, University of Rostock, 18051 Rostock, Germany; benjamin.beichler@uni-rostock.de (B.B.); christian.haubelt@uni-rostock.de (C.H.); 2 Institute of Computer Science, University of Rostock, 18051 Rostock, Germany; albert.hein@uni-rostock.de (A.H.); thomas.kirste@uni-rostock.de (T.K.)

**Keywords:** piecewise linear approximation, segmentation, sensor data abstraction, sensor signal compression, CPLR, wireless sensor networks, embedded sensor processing

## Abstract

Piecewise linear approximation of sensor signals is a well-known technique in the fields of Data Mining and Activity Recognition. In this context, several algorithms have been developed, some of them with the purpose to be performed on resource constrained microcontroller architectures of wireless sensor nodes. While microcontrollers are usually constrained in computational power and memory resources, all state-of-the-art piecewise linear approximation techniques either need to buffer sensor data or have an execution time depending on the segment’s length. In the paper at hand, we propose a novel piecewise linear approximation algorithm, with a constant computational complexity as well as a constant memory complexity. Our proposed algorithm’s worst-case execution time is one to three orders of magnitude smaller and its average execution time is three to seventy times smaller compared to the state-of-the-art Piecewise Linear Approximation (PLA) algorithms in our experiments. In our evaluations, we show that our algorithm is time and memory efficient without sacrificing the approximation quality compared to other state-of-the-art piecewise linear approximation techniques, while providing a maximum error guarantee per segment, a small parameter space of only one parameter, and a maximum latency of one sample period plus its worst-case execution time.

## 1. Introduction

Within the last two decades, different Piecewise Linear Approximation (PLA) techniques, sometimes also called Segmentation Algorithms, have been developed and studied by different researchers. The applications include data mining [1], data collection in wireless sensor networks [2,3], and several activity recognition applications with wearable sensor nodes [4,5,6]. The reasons are two-fold: Firstly, approximating sensor signals with linear segments creates an alternative representation of the characteristic shape of that signal. This is exploited by classification approaches like motif discovery [6] or trajectory-based distance metrics [4,7]. Secondly, a reduced amount of data is required to represent a signal and to process it further. This is important for data mining when huge amounts of data have to be processed. Furthermore, in activity recognition settings with wearable sensor nodes, data collected at sampling frequencies of e.g., 100 Hz has either to be stored for longer periods of time or transmitted via wireless interfaces. The motivation to reduce sensor data already on the wearable sensor node results from the significant energy consumption introduced by either transmitting raw sensor data wirelessly [5,8] or by storing it to flash memory for later offline processing [9]. Both consumes a significant amount of energy on wearable sensor nodes. Since PLA techniques allow a reduction of sensor data while providing a signal representation that has shown to be well suited for activity recognition and data mining purposes, our focus within this paper is on PLA algorithms.

While the most optimized state-of-the-art PLA algorithm [5] has been designed for embedded wearable devices with limited processing power, it still needs a buffer for sensor data in order to achieve a good compression. Such buffer-based PLA techniques can only produce segments with a maximum length of the buffer size. In situations where the sensor signal has no fluctuations (e.g., an accelerometer which is not moved over a longer period), many segments as small as the buffer size would be created instead of a single long segment. Therefore, the segment’s length is constrained by the memory budget of the architecture. Furthermore, the execution time of most state-of-the-art PLAs depends on the segments’ lengths. This in turn requires a small buffer or a limit on the segments’ length to fulfill possibly existing real-time requirements. Limiting the buffer size in turn decreases the possible compression and thus the reduction of energy consumption due to wireless transmissions.

In the context of activity recognition, a PLA algorithm has the following constraints when performed on a wireless sensor node:

- a maximum segment error guaranty must be ensured,
- an execution time that is guaranteed to be smaller than the sampling period in order to fulfill real-time requirements,

while targeting the objectives:

- minimal memory consumption,
- low latency of segment creation,
- high compression ratio, and
- small average computation time, preventing high energy consumption of the processing unit.

Although many online PLA approaches have been reported in literature for compressing sensor data or time series data in general [1,4,10,11] and even some of them already introduced PLA approaches to reduce energy consumption in WSNs (Wireless Sensor Networks) [2,3,5], none of them can fulfill the all constraints without compromising the objectives.

To the best of our knowledge, our proposed algorithm introduced by the paper at hand is the only existing PLA algorithm that offers both a computational complexity of O(1) and a memory complexity of O(1). Moreover, our evaluations for an ARM Cortex-M4 microcontroller showed a maximum execution time of at most 203 instructions per invocation, while having a memory requirement of only 23 variables. This is a considerable reduction when compared to state-of-the-art PLA algorithms. Furthermore, our algorithm provides comparable approximation quality to existing solutions, a maximum least-squares error guarantee per segment, as well as a low latency and a small parameter space.

Our paper is structured as follows. In Section 2, related work is discussed and differences to our approach are elaborated. This is followed by a thorough description of the mathematical basics to reproduce our algorithm in Section 3. In Section 4, our algorithm is explained in detail, including a version for multi-dimensional signals. An implementation of our algorithm is compared to two other existing state-of-the-art PLA techniques in Section 5, which is followed by a discussion of basic properties of our algorithm in Section 6. Conclusions are drawn in Section 7.

## 2. Related Work

There are several online PLA algorithms available in literature, also referred to as segmentation algorithms. In [1], the SWAB (Sliding Window and Bottom Up) algorithm has been introduced, which is a combination of the offline Bottom-Up segmentation and the Sliding Window (SW) approach, to create an online algorithm. A conceptual optimization of this algorithm has been introduced in [4] called mSWAB, which was further improved for resource constrained wearable sensor nodes in [5], called emSWAB. All the aforementioned approaches require to buffer data, which in turn constrains the segment length with the maximum buffer size. Furthermore, even including the most optimized version emSWAB, the aforementioned approaches have worst-case execution times depending on the length of the current segment within the buffer. This results from the iterative computation of the residual error of that segment, which has to be completely recalculated when a new sample is added. In contrast, our proposed approach has a small and data independent worst-case execution time, especially regarding the current segment’s length, as, for a new sensor sample, the segment and its error can be iteratively updated without the need of iterating over all samples covered by that segment again.

Furthermore, all the aforementioned approaches are based on *linear interpolation*, while our approach is based on *linear regression*. In [1], it was reported that linear regression based segmentations of time series lead to a disjoint look of the approximated signal since the segments are not connected. In the paper at hand, we show that this can be avoided by calculating a linear regression without an intercept term, allowing a segment originating in the previous segment’s endpoint.

In [2,3], PLA algorithms have been introduced to reduce the amount of data collected in WSNs by approximating the signals. Both algorithms are buffer-based with a worst-case computation time depending on the buffer length as well. In [10], a fast alternative for time series segmentation is reported, which trades computation time with a drastic increasing memory consumption. The authors argue “that we will soon have infinite storage so that trading storage for speed is a good choice” [10]. This obviously does not apply for resource constrained architectures like wearable sensor nodes. In contrast, our proposed approach has a constant computation and memory complexity at the same time.

In [11], another segmentation approach based on Polynomial Least-Squares Approximation with polynomials of arbitrary order is introduced. Since these also include first order polynomials that leads to linear segments, they need to be considered here as well. While their approach leads to a fast computation time for each new data point independent of the segment’s length, they still need to buffer data, if a piecewise linear approximation is targeted, which requires both segmentation points and slopes. In their experiments, it can be seen that their approach using first order polynomials basically leads to similar results with respect to approximation error and compression rate when compared to SW.

Another PLA algorithm with a constant update time has been introduced in [12]. However, again their approach is buffer-based as well with a worst-case space complexity of O(n) and uses in their experiments around 1 KB memory. Furthermore, their approach is a mixture between connected and non-connected piecewise segments. With our algorithm, we address the problem of approximating sensors signals with connected piecewise segments, which is fast and small enough to be performed on processing and memory constrained architectures.

## 3. Simple Linear Regression

Before describing our approach, we want to recap some basics on *simple linear regression* first. Simple linear regression is a linear regression model that explains the relation of a dependent variable as a function of an independent variable as best in terms of the minimum sum of squared residuals (*SSR*) error. Usually, the residuals are minimized by using the ordinary least squares method. The linear model, in the following referred to as *regression line*, is usually described as y=α+βx, with α being the intercept term with the *y*-axis and β being the slope of the regression line. As we focus on a signal approximation by connected linear segments, we force the regression line to pass through the origin. This results in a regression line without intercept term α:

(1) y=βx.

In general, it is not possible to model a set of *n* observed data pairs $\left\{\left(x_{1},y_{1}\right),\left(x_{2},y_{2}\right),...,\left(x_{n},y_{n}\right)\right\}$ with a single value $\beta_{n}$. Instead of deviations from this model, called errors, can be observed. The underlying relationship between $y_{i}$ and $x_{i}$ including the corresponding errors $e_{i}$ is described by:

(2) $$y_{i}=\beta_{n}x_{i}+e_{i}.$$

For a given estimator ${\hat{\beta }}_{n}$, the SSR error is thus computed by:

(3) $$SSR_{n}=\sum_{i=1}^{n}{\left(y_{i}-{\hat{\beta }}_{n}x_{i}\right)}^{2}=\sum_{i=1}^{n}e_{i}^{2}.$$

For the general case of linear regression with multiple independent variables, where *Y* is the vector of responses $y_{i}$ for all i=1,⋯,n, *X* is a so called design matrix with each row being the observation vector $x_{ij}$ with i=1,⋯,n and columns *j* for each independent variable (regressors). For this case, $\beta_{n}$ is a vector comprising the parameters of each regressor. Note that here the constant term is not modeled explicitly, but included in the design matrix as one column $x_{i1}=1$ for all i=1,⋯,n. Thus, the parameter $\beta_{1_{n}}$ is the resulting intercept term. The well-known ordinary least squares (OLS) estimator ${\hat{\beta }}_{n}$ is:

(4) $$\hat{\beta_{n}}={\left(X^{T}X\right)}^{-1}X^{T}y={\left(\frac{1}{n}\sum_{i=1}^{n}x_{i}x_{i}^{T}\right)}^{-1}\cdot \frac{1}{n}\sum_{i=1}^{n}x_{i}y_{i}\;,$$

with ${\left(X^{T}X\right)}^{-1}X^{T}$ being the *Moore-Penrose pseudoinverse* of *X*. From Equation (4), it can be seen that, in simple linear regression with only one independent variable and without the intercept term, $x_{i}$ is a scalar and the scalar estimator ${\hat{\beta }}_{n}$ is calculated by:

(5) $${\hat{\beta }}_{n}=\frac{\sum_{i=1}^{n}x_{i}y_{i}}{\sum_{i=1}^{n}x_{i}^{2}}=\frac{{\bar{xy}}_{n}}{{\bar{x^{2}}}_{n}},$$

with ${\bar{xy}}_{n}=\frac{\sum_{i=1}^{n}x_{i}y_{i}}{n}$ and ${\bar{x^{2}}}_{n}=\frac{\sum_{i=1}^{n}x_{i}^{2}}{n}$ denoting the mean of all $x_{i}y_{i}$ products and the mean of all $x_{i}$ squares up to the *n*-th observation, respectively.

In Section 4, we show that this allows us to quickly determine ${\hat{\beta }}_{n}$ for a new data point by updating ${\bar{xy}}_{n}$ and ${\bar{x^{2}}}_{n}$. In order to calculate the $SSR_{n}$ from the updated variables and the OLS estimator ${\hat{\beta }}_{n}$ for each new data point, we need to resolve Equation (3) with the binomial formula to:

(6) $$SSR_{n}=\sum_{i=1}^{n}y_{i}^{2}-2{\hat{\beta }}_{n}\sum_{i=1}^{n}x_{i}y_{i}+{\hat{\beta }}_{n}^{2}\sum_{i=1}^{n}x_{i}^{2}.$$

Each sum can then be rewritten using the corresponding mean values multiplied by *n*:

(7) $$SSR_{n}={\bar{y^{2}}}_{n}n-2{\hat{\beta }}_{n}{\bar{xy}}_{n}n+{\hat{\beta }}_{n}^{2}{\bar{x^{2}}}_{n}n.$$

Resolving ${\hat{\beta }}_{n}^{2}{\bar{x^{2}}}_{n}$ to ${\hat{\beta }}_{n}{\bar{xy}}_{n}$ from Equation (5) gives:

(8) $$SSR_{n}=n\left({\bar{y^{2}}}_{n}-{\hat{\beta }}_{n}{\bar{xy}}_{n}\right).$$

### Incremental Updating

By using Equation (5) for calculating the slope and Equation (8) for calculating the error, all the necessary means ${\bar{x^{2}}}_{n}$, ${\bar{xy}}_{n}$, and ${\bar{y^{2}}}_{n}$ up to the *n*-th sample can be updated and the corresponding ${\hat{\beta }}_{n}$ and $SSR_{n}$ can be recalculated with a constant computational complexity independent of *n* for each new data point. This is based on updating a mean ${\bar{z}}_{n}$ with each new sample $z_{i}$ by:

(9) $${\bar{z}}_{n}=\frac{\sum_{i=1}^{n}z_{i}}{n}=\frac{\sum_{i=1}^{n-1}z_{i}+z_{n}}{n}.$$

Substituting $\sum_{i=1}^{n-1}z_{i}$ with ${\bar{z}}_{n-1}\cdot \left(n-1\right)$ gives the equation updating the mean ${\bar{z}}_{n}$ with the new sample $z_{n}$ from [13]:

(10) $${\bar{z}}_{n}=\frac{{\bar{z}}_{n-1}\left(n-1\right)}{n}+\frac{z_{n}}{n}.$$

We rearranged the equation to:

(11) $${\bar{z}}_{n}={\bar{z}}_{n-1}+\frac{z_{n}-{\bar{z}}_{n-1}}{n},$$

which sacrifices slightly in numerical precision, which will be discussed in Section 6.1, but saves a multiplication and a division, in order to reduce the processing time.

This equation can be used for updating all means ${\bar{x^{2}}}_{n}$, ${\bar{xy}}_{n}$, and ${\bar{y^{2}}}_{n}$ for each new sample to recalculate ${\hat{\beta }}_{n}$ and $SSR_{n}$ with Equations (5) and (8) in constant time, respectively.

## 4. Online Piecewise Linear Approximation

We now introduce a problem definition of online piecewise linear approximation of sensor signals. Assume a sensor signal sampled at different timestamps, which leads to a series *S* of sensor samples $\left(\left(s\left[1\right],\tau_{s}\left[1\right]\right),\left(s\left[2\right],\tau_{s}\left[2\right]\right),\cdots ,\left(s\left[n\right],\tau_{s}\left[n\right]\right)\right)$ where each sample $\left(s,\tau_{s}\right)$ consists of the signal value *s* and the corresponding timestamp $\tau_{s}$. A piecewise linear approximation of that signal is a series $\tilde{S}$ of segment points $\left(\left(\tilde{s}\left[1\right],\tau_{\tilde{s}}\left[1\right]\right),\left(\tilde{s}\left[2\right],\tau_{\tilde{s}}\left[2\right]\right),\cdots ,\left(\tilde{s}\left[n\right],\tau_{\tilde{s}}\left[n\right]\right)\right)$ where each segment point $\left(\tilde{s},\tau_{\tilde{s}}\right)$ consists of a signal value $\tilde{s}$ and its corresponding timestamp $\tau_{\tilde{s}}$ and represents the end point of the previous segment and the start point of the next segment. A segment is thus represented by a pair of consecutive segment points $\left(\left(\tilde{s}\left[k-1\right],\tau_{\tilde{s}}\left[k-1\right]\right),\left(\tilde{s}\left[k\right],\tau_{\tilde{s}}\left[k\right]\right)\right)$. Furthermore, $\forall \tau_{\tilde{s}}\left|\left(\tilde{s},\tau_{\tilde{s}}\right)\epsilon \tilde{S}:\exists \left(s,\tau_{s}\right)\epsilon S\right|\tau_{s}=\tau_{\tilde{s}}$ holds, as the segmentation points are created at timestamps of the original sensor samples. Additionally, a user-defined threshold TH on the SSR of a segment is given, which needs to be guaranteed for all segments within the approximation process.

**Figure 1:**
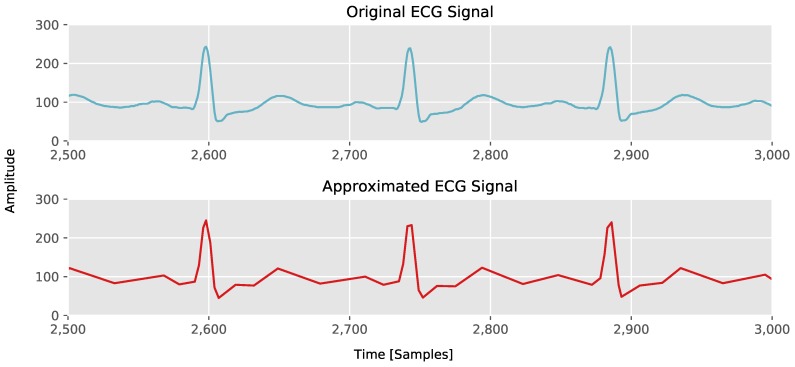
Part of ECG signal from Section 5.1 approximated with our Connected Piecewise Linear Regression (CPLR) algorithm.

An example of a piecewise linear approximated Electrocardiography (ECG) signal can be seen in Figure 1.

Some PLA techniques use, for the approximation of a segment, the original sample values at the beginning and ending of the segment. Similar to [12], our approach does also use approximations of those values. We can not draw any advantage or disadvantage from this characteristic as can be seen in Section 5. With growing segment sizes, the ratio of original values to approximated values would become fairly small, whereby the influence additionally decreases. Since the approximation of the segment points’ values are included in the SSR calculation, the TH is guaranteed to be an upper bound on the approximation error of a segment.

In order to achieve a piecewise linear approximation $\tilde{S}$ of a sensors signal *S* online, the sensor signal needs to be continuously represented, i.e., the segment corresponding to the last sensor sample needs to be outputted as soon as its end point is determined due to an exceeded threshold. Therefore, online PLA algorithms perform a sample-based processing, which means that the algorithm is invoked with each new sensor sample. The processing time for each invocation of the algorithm is referred to as its execution time.

In the process of online approximation, the average execution time needs to be smaller than the sampling period, in order to not lose any samples. In order to avoid additional latencies and additional memory consumption due to input buffering, even the worst-case execution should be smaller than the sampling period. Moreover, a data independent execution time is preferable, as this guarantees a predictable maximum sampling period at which the algorithm is able to approximate the signal in real time without compromising the compression abilities.

The SW algorithm for example adds a new sample to the current segment in its buffer, each time the algorithm is invoked. It creates a segment from the previous segment’s end point to the newly added sample and calculates the residual error of this segment. This error calculation involves *n* steps for a segment length of *n* and has to be recalculated each time the algorithm is invoked, as the segment changes with each newly added sample. If the calculated segment error is below TH, the routine returns without outputting that segment and will be invoked with the next incoming sensor sample. If the segment error is above TH, the segment from the previous invocation is recreated, which still satisfied the maximum error guarantee and is outputted. A new segment is started from this point. Due to the error calculation, the execution time of the SW algorithm depends on the current segment’s length and increases with each invocation in which the segment error has not reached TH. Furthermore, the memory consumption for buffering the last *n* sensor samples increases likewise. Thus, with longer segments, both the execution time and the memory consumption of each invocation increases. In order to guarantee a maximum execution time and memory consumption, the maximum buffer size needs to be constrained to a possibly small size, which in turn limits the compression ability of the algorithm.

### 4.1. CPLR Approximation

In order to overcome this drawback, our algorithm makes use of the updating abilities of simple linear regression as discussed in Section 3. Instead of recalculating the slope and the error between the previous segment’s endpoint and the current sensor sample, our approach updates the slope ${\hat{\beta }}_{n}$ and the corresponding segment error ($SSR_{n}$) of the regression line with each new sensor sample. Since the regression line has its origin in the previous segment point, our algorithm is performing a Connected Piecewise Linear Regression (CPLR) of the sensor signal.

This regression line and its error can be updated by a small number of calculations shown in the previous section, independent of the segment’s length, while obtaining the minimal SSR to the original data. The idea is as follows: the (k-1)-th segment , which is the regression line of a simple linear regression, starts at the end point of the previous segment $\left(\tilde{s}\left[k-1\right],\tau_{\tilde{s}}\left[k-1\right]\right)$. Thus, ${\bar{x^{2}}}_{n}$, ${\bar{y^{2}}}_{n}$, and ${\bar{xy}}_{n}$ are updated by Equation (11) with $y_{n}$ being the difference between a new sample’s value s[n] and the value of the previous segment point $\tilde{s}\left[k-1\right]$ and $x_{n}$ being the difference between the new sample’s timestamp $\tau_{s}\left[n\right]$ and the previous segment point’s timestamp $\tau_{\tilde{s}}\left[k-1\right]$.

Note that we do not require the sensor signal to be sampled at equidistant intervals for our approach. However, without loss of generality, we will presume a constant sampling period in the following for the sake of simplicity. Thus, the sample number *n* within the current segment can be used for the $x_{n}$ values instead. The pseudo code for our CPLR routine is given in Algorithm 1.

**Algorithm 1** Connected Piecewise Linear Regression.
1:**procedure**process_sample(sample value *s*, segment array $\tilde{S}\left[\right]$, index *k*)2:   n=n+13:   $y=s-value(\tilde{S}\left[k-1\right])$4:   ${\bar{x^{2}}}_{n}={\bar{x^{2}}}_{n-1}+\left(\left(n\cdot n\right)-{\bar{x^{2}}}_{n-1}\right)/n$5:   ${\bar{xy}}_{n}={\bar{xy}}_{n-1}+\left(\left(n\cdot y\right)-{\bar{xy}}_{n-1}\right)/n$6:   ${\bar{y^{2}}}_{n}={\bar{y^{2}}}_{n-1}+\left(\left(y\cdot y\right)-{\bar{y^{2}}}_{n-1}\right)/n$7:   ${\hat{\beta }}_{n}={\bar{xy}}_{n}/{\bar{x^{2}}}_{n}$8:   $SSR_{n}=\left({\bar{y^{2}}}_{n}-{\hat{\beta }}_{n}{\bar{xy}}_{n}\right)\cdot n$9:   **if** $SSR_{n}<=TH$ **then**10:      ${\bar{x^{2}}}_{n-1}={\bar{x^{2}}}_{n}$11:      ${\bar{xy}}_{n-1}={\bar{xy}}_{n}$12:      ${\bar{y^{2}}}_{n-1}={\bar{y^{2}}}_{n}$13:      ${\hat{\beta }}_{n-1}={\hat{\beta }}_{n}$14:      return15:   16:   $t=timestamp(\tilde{S}\left[k-1\right])+n-1$17:   $\tilde{s}=value\left(\tilde{S}\left[k-1\right]\right)+\left({\hat{\beta }}_{n-1}\cdot \left(n-1\right)\right)$18:   n=119:   $y=s-\tilde{s}$20:   ${\bar{x^{2}}}_{n}=1$21:   ${\bar{xy}}_{n}=y$22:   ${\bar{y^{2}}}_{n}=y\cdot y$23:   ${\hat{\beta }}_{n}={\bar{xy}}_{n}/{\bar{x^{2}}}_{n}$24:   $SSR_{n}=0$25:   ${\bar{x^{2}}}_{n-1}={\bar{x^{2}}}_{n}$26:   ${\bar{xy}}_{n-1}={\bar{xy}}_{n}$27:   ${\bar{y^{2}}}_{n-1}={\bar{y^{2}}}_{n}$28:   ${\hat{\beta }}_{n-1}={\hat{\beta }}_{n}$29:   $\tilde{S}\left[k\right]=\left(\tilde{s},t\right)$30:   return


The threshold value TH is set upon initialization and needs to be stored globally. When starting the online approximation of a sensor signal, the signal’s very first sample $\left(s\left[1\right],\tau_{s}\left[1\right]\right)$ will be used as the initial segment point $\left(\tilde{s}\left[1\right],\tau_{\tilde{s}}\left[1\right]\right)=\left(s\left[1\right],\tau_{s}\left[1\right]\right)$. The routine *PROCESS_SAMPLE* is called for each new sampled sensor value *s*, which is the first parameter given to the function along with the array $\tilde{S}\left[\right]$ for storing segments and the index *k* for specifying at which position in $\tilde{S}\left[\right]$ the new segment will be stored. Note that the array of segments $\tilde{S}\left[\right]$ does not necessarily need to store all the created segments, e.g., when always transmitting each new segment point to another device. However, $\tilde{S}\left[\right]$ needs a size of at least two: for the previous segment’s end point and the current segment’s endpoint, which will be written to it when created. The variables *n*, ${\bar{x^{2}}}_{n-1}$, ${\bar{xy}}_{n-1}$, ${\bar{y^{2}}}_{n-1}$, and ${\hat{\beta }}_{n-1}$ need to be stored globally and must be initialized with zero before starting the online approximation of a sensor signal.

At the beginning, the size *n* of the current segment is incremented by the new sample in line 2. In line 3, the actual *y* value is calculated from the new sensor value *s* and the previous segment points value $\tilde{s}\left[k-1\right]$. The function *value()* returns the value part and *timestamp()* returns the timestamp part of the segment point $(\tilde{s}\left[k-1\right],\tau_{\tilde{s}}\left[k-1\right]),$ respectively. From lines 4 to 8, the means ${\bar{x^{2}}}_{n}$, ${\bar{xy}}_{n}$, and ${\bar{y^{2}}}_{n}$ are updated from their previous values ${\bar{x^{2}}}_{n-1}$, ${\bar{xy}}_{n-1}$, and ${\bar{y^{2}}}_{n-1}$ with the new *y* value, respectively, and the estimated slope ${\hat{\beta }}_{n}$ and the new segment error $SSR_{n}$ is calculated. If the new $SSR_{n}$ is below TH (line 9), the updated means ${\bar{x^{2}}}_{n}$, ${\bar{xy}}_{n}$, and ${\bar{y^{2}}}_{n}$ and the slope ${\hat{\beta }}_{n}$ are stored for the next invocation (line 10 to 13) and the routine returns without creating a new segment point. If $SSR_{n}$ exceeds TH instead, a new segment point is created at the previous sample’s timestamp (line 16) by extrapolating the value of the segment’s endpoint from the previous invocation’s slope estimate ${\hat{\beta }}_{n-1}$ (line 17) with the following equation:

(12) $$\tilde{s}\left[k\right]={\hat{\beta }}_{n-1}\left(n-1\right)+\tilde{s}\left[k-1\right].$$

**Figure 2:**
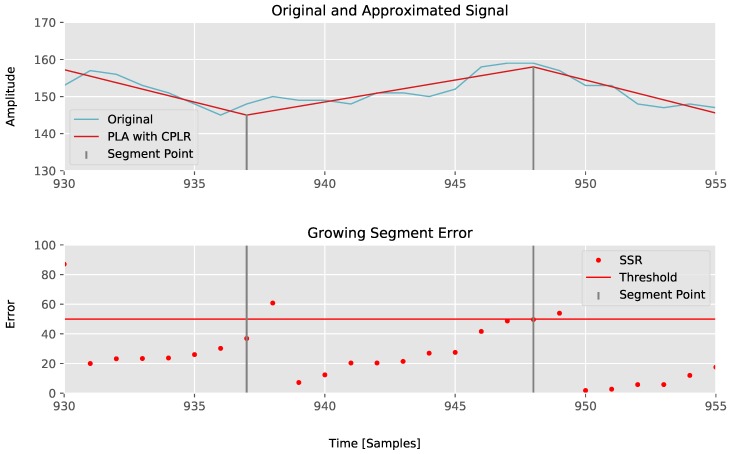
Growing sum of squared residuals error per sample.

The newly started segment’s ($\tilde{s}\left[k+1\right]$) size is updated in line 18, and the *y* value from the sample *s* to the newly created segment’s value $\tilde{s}$ is calculated in line 19. From lines 20 to 23, the means are initialized with the new *y* as the first sample of the newly started segment and the slope ${\hat{\beta }}_{n}$ is recalculated from them. The $SSR_{n}$ of the new segment is set to zero, as the new segment only consists of two values, which introduces no deviations. From lines 25 to 28, the means ${\bar{x^{2}}}_{n}$, ${\bar{xy}}_{n}$, and ${\bar{y^{2}}}_{n}$ and ${\hat{\beta }}_{n}$ are stored and finally the new segment point $\left(\tilde{s},t\right)$ is written to the segments array in line 29. For the sake of clarity, the principle including the $SSR_{n}$ exceeding TH is illustrated in Figure 2.

Note that the error metric per segment can easily be changed to an average per sample approximation error metric by dividing the current segment’s $SSR_{n}$ error by the number of samples *n* covered by that segment. This can be useful in applications that require a guaranteed relative least-squares approximation error bound. Furthermore, an average per sample approximation error allows for specifying a maximum error guarantee for a finite set of samples, if TH is chosen to be the maximum error divided by the number of samples.

### 4.2. Multi-Dimensional CPLR Implementation

The algorithm can be implemented to approximate multi-dimensional signals with *D* dimensions, by holding and updating the variables ${\bar{xy}}_{n}^{d}$, ${\bar{y^{2}}}_{n}^{d}$, ${\hat{\beta }}_{n}^{d}$, and $SSR_{n}^{d}$ respectively for each dimension *d* and the mean of the timestamps ${\bar{x^{2}}}_{n}$ for all dimensions together, as the value of each dimension is supposed to be sampled at the same time. As we use the sum of squared residuals error, the error of the resulting *D*-dimensional segment can be calculated by summing up the $SSR_{n}^{d}$ of all dimensions. This is possible due to the commutative property of summing up the squared residuals:

(13) $$\sum_{i=1}^{n}\sum_{d=1}^{D}{\left(y_{i}^{d}-{\hat{\beta }}_{n}^{d}x_{i}^{d}\right)}^{2}=\sum_{d=1}^{D}\sum_{i=1}^{n}{\left(y_{i}^{d}-{\hat{\beta }}_{n}^{d}x_{i}^{d}\right)}^{2}.$$

Thus, the residual error to the *D*-dimensional segment is calculated by:

(14) $$SSR_{n}=\sum_{d=1}^{D}SSR_{n}^{d}.$$

Note, that using the sum of squared residuals error metric leads to a faster calculation on resource constraint architectures, as no square root has to be calculated. However, other solutions to calculate a multidimensional error are possible as well, e.g., sum of Euclidean distances.

## 5. Evaluation

In order to show the approximation quality of CPLR, we compared it to two other piecewise linear approximation techniques from the literature. We decided to use the emSWAB implementation from [5], as it was specifically designed for resource constrained architectures and we implemented the SW algorithm as well. Since the emSWAB implementation is made for 8 bit data, we implemented our CPLR algorithm and the SW approximation for 8 bit data as well to stay comparable, while internally using single precision floating point data types for the variables like slope, error, and means. This is a fair comparison as all implementations were tested on an x86-64 system, from which the emSWAB implementation benefits in the same way as our implementations, as emSWAB does not save intermediate results for slope and error calculation in 8-bit variables. The algorithms for our experiments are implemented in C. Our implementations can be obtained by mailing the paper’s first or last author. For the following experiments, we used emSWAB with a buffer size of 50 and SW with a buffer size of 100. This is a fair comparison, as the emSWAB algorithm actually scales the buffer size between half and double of the specified value. This means, in the following experiments, the emSWAB’s maximum buffer size is 100 as well.

### 5.1. Data Sets

**Figure 3:**
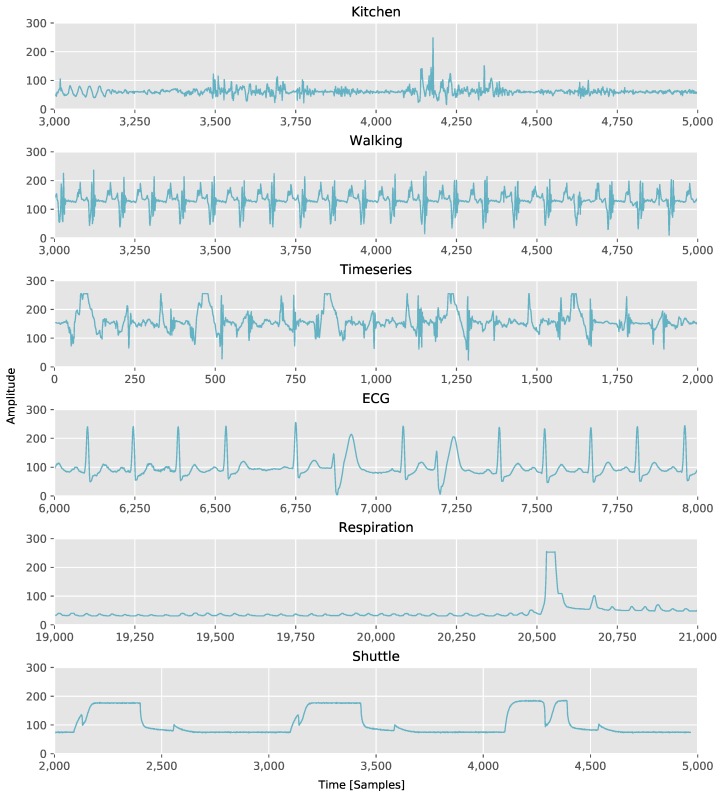
Partially plotted data sets for evaluation.

As the approximation quality of the algorithms depends on the actual data, we used different data sets for the evaluation. In our first test, we recorded accelerometer and gyroscope data with a wireless sensor node in two scenarios. In the *Kitchen* scenario, the sensor was attached to the wrist of a user, while the user performed kitchen tasks like cutting carrots with a knife, stirring a bowl of ingredients with a wooden spoon, blending ingredients in a bowl with a hand-held blender and using a hand mixer. For both data (accelerometer and gyroscope), we used the vector lengths of the three-dimensional signals, in order to derive one-dimensional signals. An example of the accelerometer signal is partially shown in Figure 3.

In a second scenario, we recorded accelerometer and gyroscope signals of a *Walking* person. The sensor was attached to the shoe of the user and sampled the data at 100 Hz. The walking dataset includes straight paths, turns, walking upstairs and downstairs and also different walking speeds. An example of the accelerometer signal is partially shown in Figure 3 as well.

As in the literature PLA algorithms are not only used for activity recognition scenarios, we also included experiments on available data sets from literature, which were used for the evaluation of PLA algorithms in other application domains. We used the timeseries data set (*Timeseries*) that came with the implementation of mSWAB from [4] as well as the data sets from [14] including ECG signals (*ECG*), valve time series of a Marotta Space Shuttle (*Shuttle*), and the time series of a patient’s respiration measured by the thorax extension (*Respiration*), which are freely available at [15]. These datasets are shown in Figure 3 as well.

### 5.2. Approximation Quallity

We found two criteria that are the most important to describe the approximation quality of a PLA algorithm: the compression ratio and the resulting approximation error per sample, which is referred to as *average residual error* in the following. Note that the average residual error needs to be distinguished from the segment error. The latter is part of the PLA algorithms for calculating the absolute residual error of a segment (SSR for CPLR), in order to guarantee a maximum error per segment, while the average residual error is used for evaluation purposes: when a data set is approximated with one of the PLA algorithms, the average sum of squares error per sample of the approximated signal to its original signal is calculated, in order to evaluate the overall approximation error of that approximation. The average residual error of an approximated signal is its SSR to the original signal, divided by the number of samples of that data set.

At different compression ratios, the average residual error differs accordingly. Although we cannot assume monotonicity, as a general trend, the more the average residual error increases, the more the data is compressed. In general, we want a small average residual error, while simultaneously achieving a high compression ratio or a low *inverse compression ratio* (ICR), respectively. Thus, these two criteria are conflicting.

At a certain threshold, a PLA algorithm produces an approximation with an average residual error and an ICR. We refer to this pair of error and ICR as an *operating point*. While CPLR uses the same segment error metric like SW (i.e., SSR), the authors of emSWAB decided to implement their segment error metric as the sum of absolute distances, in order to avoid computationally costly square and square-root functions. However, this causes a different behavior on the threshold. While CPLR and SW reach an operating point at a certain threshold, emSWAB leads to a higher approximation error, but also higher compression ratio at the same threshold, thus another operating point. The interesting question is whether this operating point is also met by SW and CPLR at a higher threshold, or whether their approximation error is smaller or higher at the same compression ratio. Thus, it is not sufficient to compare two algorithms with the same threshold in order to compare the approximation quality. Both algorithms might lead to the same operating point at different thresholds. Furthermore, as the approximation of a time series for different thresholds is a discrete problem, it is not always possible to find a threshold, which leads to the exact same ICR or average residual error, respectively, of two PLA algorithms. In order to compare different PLA algorithms, we approximated the same time series multiple times with different thresholds and plotted the the resulting average residual error over the corresponding ICR (amount of compressed data to the amount of uncompressed data) for each operating point controlled by the threshold. This leads to curves sketching the dependency between both quality indicators of the PLA algorithms. Note that we used the ICR instead of the compression ratio, as it gives an intuition about the resulting relative data size after approximating a sensor signal. The nearer the curve is to the origin of the plot, the better is the approximation quality of the algorithm.

In our experiments, we evaluated 29 data sets with in total 536,175 samples, all of them approximated 10,000 times with emSWAB from threshold 1 to 10,000 and 100,000 times with both SW and CPLR from threshold 1 to 100,000. The range of thresholds at which emSWAB was performed in our experiments is smaller than for CPLR and SW, as the error metric of emSWAB is a sum of absolute distances. This is explained in the following. At a certain threshold, the emSWAB algorithm might lead to an operating point that can be achieved with CPLR and SW at a much higher threshold, since the sum of squares error in CPLR and SW reaches the threshold faster than the sum of absolute distances used in emSWAB. Thus, the range of thresholds at which CPLR and SW were evaluated in our experiments needed to be larger as for emSWAB in order to reach the same range of operating points for comparisons. Note, that the segment error is an absolute error for a whole segment while the average residual error is a per-sample error. Thus, the segment errors and correspondingly the thresholds are higher then the resulting average residual error in the plots.

**Figure 4:**
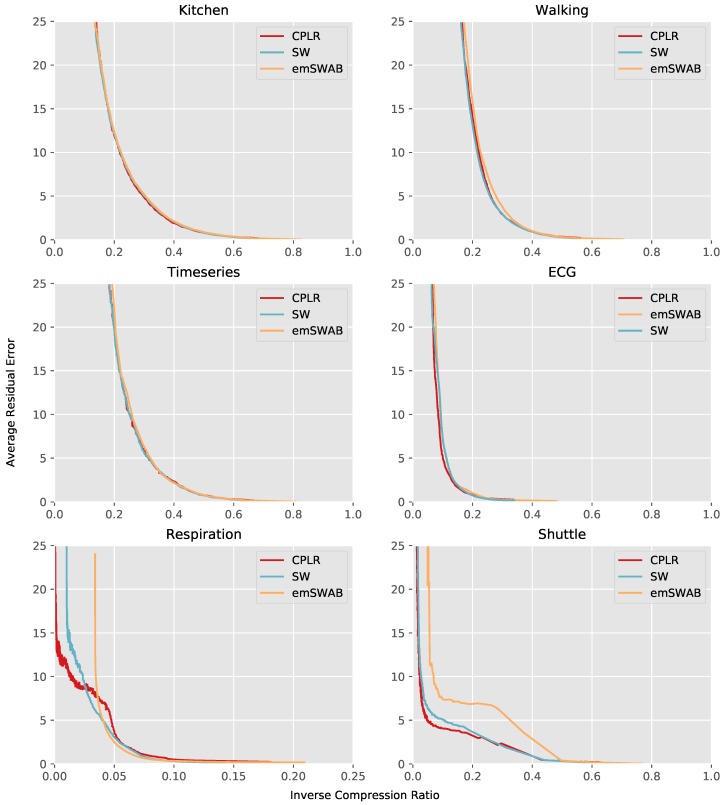
Evaluation of different data sets.

As can be seen in Figure 4, the general trend is a higher average residual error for lower ICRs. Comparing the quality plots *Kitchen*, *Walking*, and *Timeseries* with *ECG*, the ICR of the latter is smaller at similar average residual errors. This means for the *ECG* data set a higher compression rate for comparable approximation errors can be achieved. This mainly results from the signal structure of the *ECG* signal, which contains a higher amount of non-fluctuating signal parts compared to the other data sets.

**Figure 5:**
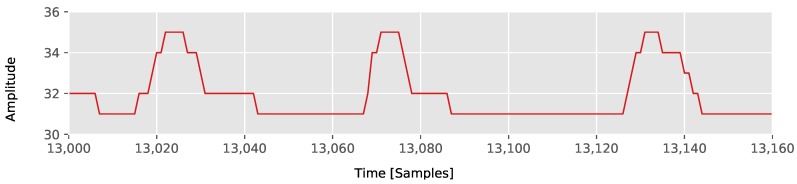
Over quantization of partially plotted Respiration signal.

For the respiration data set, the compression ability appears to be even higher. Note that, due to the high compression of this data set, we plotted the quality curve of the Respiration data at a smaller ICR range, in order to distinguish the curves. However, by having a closer look at the signal, the high compression results out of an over quantization of the signal when converting it to 8 bit because of the signal peak, which can be seen in Figure 3, representing the respiration of the person while waking up. The over quantization leads to a signal mostly consisting of perfect straight parts, which can be seen in Figure 5. Thus, the possible compression rate for this data set is much higher. Since for the Respiration data set a significant compression is possible, the bound on the possible achievable compression of SW due to its maximum buffer size can be observed in Figure 4. For SW, the ICR is bounded to 0.01 as a minimum, as the maximum buffer size is set to 100, while CPLR allows for unbounded segment sizes and thus a higher compression or a lower ICR, respectively.

Note that the curves in Figure 4 represent operating points of different integer threshold values starting from 1. Due to the nature of the error metrics implemented in the algorithms, the curves start at different operating points for a threshold of 1. By using floating point thresholds, the operating points below 1 could be reached as well, which are expected to follow the trend towards a lower error and a higher ICR as well. However, this is not further investigated in our experiments, as the operating points of interest with lower ICRs can already be seen in the conducted experiments. Furthermore, we did not include TH=0 in Figure 4, as it might cause misleading assumptions. In fact, CPLR as well as SW will recreate the original signal at TH=0, while compressing perfect straight parts of the signal that can be approximated by a segment without any approximation error. However, the emSWAB implementation from [5] does not behave in the same way and does not compress the signal at TH=0. This is basically caused by the implementation of emSWAB, which merges segments when the residual error is below TH instead below or equal TH, which is too conservative. However, an emSWAB implementation with the same behavior as our CPLR and SW implementations is possible, but was not available for our studies. Furthermore, the ICR at TH=0 is approx. 32% with CPLR and SW for the *Respiration* data set shown in Figure 3. This is caused by the coarse quantization of the signal. In activity recognition scenarios with MEMS (Micro-Electro-Mechanical Systems) accelerometers and gyroscopes, the signals typically use 16 bit fixed-point data or floating point data and include significant noise on the least significant bits. In such cases, we do not expect a significant compression at TH=0, and thus excluded TH=0 from our evaluation in Figure 4.

In this experiment, we compared the approximation quality of our CPLR algorithm with SW and emSWAB. In general, our evaluation shows no significant differences in the approximation quality of the chosen algorithms, except the ability of CPLR to create much longer segments and the approximation of the shuttle dataset, for which emSWAB slightly sacrifices in its approximation quality. Thus, we can show, that although CPLR is calculated in a time and memory efficient manner, its approximation quality is not compromised by this.

### 5.3. Execution Time

As a first evaluation of the execution time behavior on the introduced data sets in Section 5.1, we used the x86-64 platform, as it allows a feasible evaluation of this amount of data. We decided to use an emulative approach based on the Valgrind framework [16]. We did not include the simulation of caching effects, although our algorithm would draw profit from it, as all calculation data will fit in first level caches. We evaluated the binaries compiled with GNU C (GCC) compiler version of 7.3.0 [17] with default configuration on an x86-64 system. As an evaluation metric, we use the measured instruction count spent in the function, which is called for every new sample of an input data series. By using the instruction count, architectural effects on the timing behavior like pipelining, branch prediction, caching, out of order execution, and the processor frequency are avoided.

For these experiments, we created Callgraphs with the tool Callgrind [18] to evaluate CPLR, SW, and emSWAB over all 29 data sets using the same buffer sizes as in Section 5.2, i.e., 100 for SW and 50 for emSWAB and a threshold of 100. The minimum, maximum, and average instruction count per algorithm invocation as well as the standard deviation and the number of invocations can be seen in Table 1. The latter column only differs in the row emSWAB, as emSWAB handles the initialization of the PLA within the same function used for approximation while CPLR and SW are implemented with a separate initialization for the very first sample, for which they are not called in each data set.

**Figure 6:**
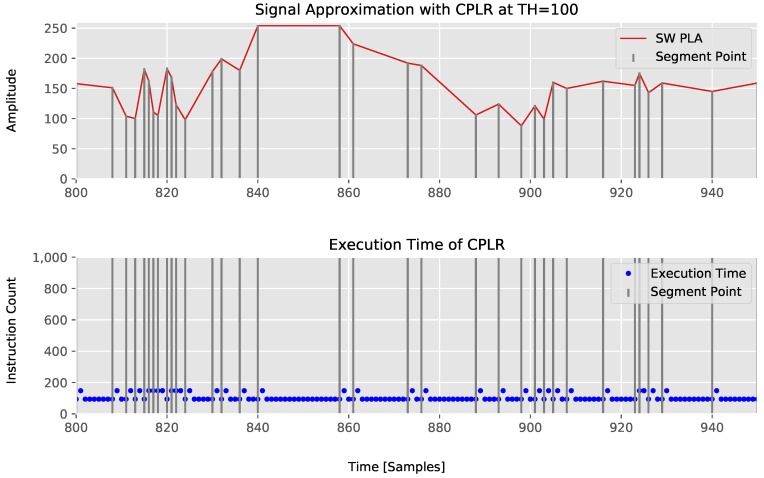
Execution time of CPLR with TH=100.

By this experiment, we can show that our CPLR algorithm has a maximum instruction count of 150. The best case is 94 instructions, which need to be executed when no segment is outputted. Note that the average instruction counts for our algorithm depends on the actual signals, as the number or length of segments respectively is decisive for the fraction and is bounded on 150 instructions in case no compression is achieved and a segment is created for each sensor sample. The execution time behavior of CPLR can be seen in Figure 6. This shows the worst-case execution time characteristic of the CPLR algorithm.

**Figure 7:**
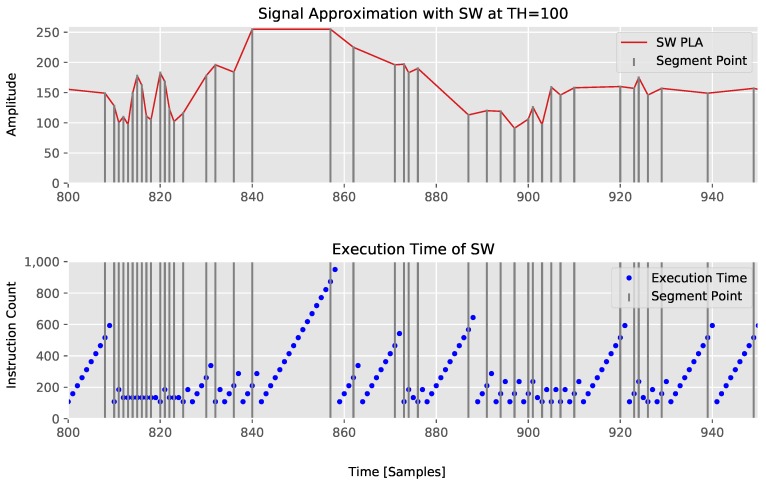
Execution time of SW with TH=100.

For SW, we observe that the instruction count of each invocation grows by approximately 51 instructions for each new sample that is added to a segment. Thus, the execution time of SW linearly increases with the current segment’s length. In the case, that a segment is created due to an exceeding threshold, 26 instructions are needed additionally to the error calculation. If a segment is created due to a maximum segment size, only 46 instructions have to be performed, because the error calculation can be skipped. Therefore, the maximum and average instruction count depends on the maximum segment’s length and can be bounded by bounding the buffer size. In Figure 7 the execution time dependency of SW on the segment’s length is shown.

**Figure 8:**
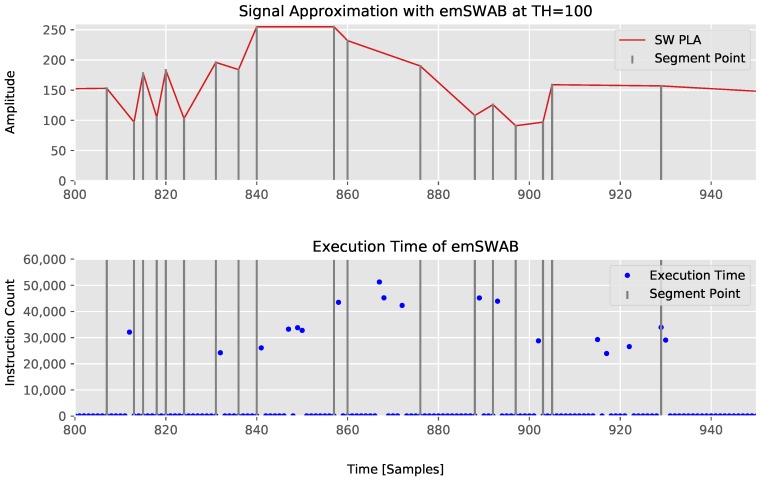
Execution time of emSWAB with TH=100.

For emSWAB, the error calculation does not happen in each invocation and mostly the new sample is simply added to the buffer. This is the best case for emSWAB with 53 instructions. The segmentation within the buffer starts when the slope sign of the signal changes, and is not necessarily correlated to segment points. In this case, the execution time drastically increases, as multiple segments within the buffer are created and merged until none of them can be merged any further due to an exceeding error threshold. Within this process, the segment error calculation similar as in SW (iterating over all samples covered by that segment) is performed but repeatedly for multiple created and merged segments. Thus, the execution time is highly data and buffer size dependent and is two orders of magnitude higher than CPLR in this experiment. The execution time behavior of emSWAB is shown in Figure 8.

From this experiment, it can be seen that our CPLR algorithm not only has the minimum average computation time, but also a minimal guaranteed maximum execution time of 150 instructions on the x86-64 architecture without compromising its compression abilities.

In order to be able to perform this experiment on a large amount and variety of signals, we used the same threshold for all algorithms on all data sets. However, this is not a fair comparison, as the algorithms might lead to different segment lengths for the same thresholds, which influences the timing behavior of SW and emSWAB. Furthermore, among different data sets, the resulting compression ratios can differ as well as the same threshold. Therefore, we performed a second experiment on one of the data sets (i.e., the ECG signal from Figure 3) and chose the thresholds for CPLR, SW, and emSWAB leading to approximately the same compression ratio. This was done by examining the results of the in total 210,000 invocations at different thresholds on the corresponding ECG data set from the quality evaluation in Section 5.2 and picking those thresholds for CPLR, SW, and emSWAB, which just achieved an inverse compression ratio of at least 0.2. The chosen thresholds, the corresponding compression ratios and the instruction counts observed in this experiment are shown in Table 2.

It can be seen that the maximum instruction count for emSWAB and SW decreased w.r.t the results in Table 1, as the lower thresholds lead to shorter segments and thus lower execution times. The average instruction count for CPLR has slightly increased, as at a lower threshold smaller and thus more segments are created. As the segment creation needs additional instructions, the average instruction count slightly increased. The maximum instruction count of 150 for CPLR does not appear in this data set. This only happens due to the truncation to 8 bit, if the approximated segment point is above 255 or below 0, which needs two instructions. Thus, the maximum instruction count of 148 in this second experiment is less than in Table 1. Furthermore, it can be seen that the average instruction count for emSWAB increased, although the maximum instruction count decreased. This is explained by the fact of a smaller threshold leading to smaller segments, and thus the computationally costly segmentation process within the buffer is faster but performed more frequently. For SW, the minimum instruction count is 57 in this experiment. This is caused by the fact that the lower threshold leads to shorter segments and no segment is created due to an exceeding maximum segment size, which needs 46 instructions. Despite the latter, an instruction count of 57 is the shortest execution time of SW, which happens when only the first sample is added to a newly created segment. This small execution time is only achieved at the very first invocation of the SW algorithm after initialization. In all other invocations, the first sample is already added to the buffer upon segment creation, since a new segment’s end point is always created for the previous sample.

#### ARM Cortex-M4 Microcontroller Architecture

On the x86-64, we were able to show the execution times of CPLR, SW, and emSWAB on a large amount and variety of data. However, as the target architectures of our algorithm are embedded microcontrollers, we additionally analyzed the instruction count on an ARM Cortex-M4 microcontroller. Due to the lack of an appropriate hardware setup on which high amounts of data can be approximated while acquiring the execution time of each invocation in an automated manner, we performed a static code analysis on the assembler code. We compiled emSWAB, SW and our CPLR algorithm for the ARM Cortex-M4 microcontroller architecture using the Arm Embedded GCC version 7.3.0 of the GNU Arm Embedded Toolchain. As compiler settings, we used Cortex-M4 as the target platform with its included hardware floating point unit. From the produced assembler code, we extracted the control flow graphs with corresponding instruction counts of each basis block. For SW and our CPLR algorithm, the simplistic control flow allows for easily analyzing the worst-case instruction count and its data dependency.

The best- and worst-case instruction counts per invocation for CPLR are 131 and 203 instructions, respectively. For SW, the best case is 59 instructions when a segment is created due to an maximum segment length. The data dependent worst case is 106+n·56 instructions with *n* being the segment size. For a maximum buffer size of 100 samples as used in the experiments on the x86-64 architecture (see Table 1), the maximum *n* is 100 and the worst-case instruction count of SW would be 5706 instructions on the ARM Cortex-M4 microcontroller. For emSWAB, we were able to analyze the shortest path consisting of 64 instructions, in which only a new sample is added to the buffer. However, when the segmentation process within the buffer is performed, the control flow consists of several nested function calls and nested loops, which makes a manual analysis infeasible. This data dependent behavior aligns with the timing behavior observed on the x86-64 architecture in our experiments (see Figure 8) and is not further investigated in this paper. The minimal and maximal instruction counts of CPLR, SW, and emSWAB on the ARM Cortex-M4 architecture are summarized in Table 3.

## 6. Discussion

Our evaluation in Section 5 shows that our CPLR algorithm achieves comparable approximation quality while having a low computational overhead compared to the state-of-the-art solutions. In the following, we want to discuss some basic properties of CPLR.

### 6.1. Numerical Precision

In general, the computations to update the mean ${\bar{x^{2}}}_{n}$, ${\bar{xy}}_{n}$, and ${\bar{y^{2}}}_{n}$ with the equations of [13] are regarded as numerically stable. However, without a buffer, our CPLR algorithm does not constrain the length of a segment by a maximum buffer size like all other existing solutions. As this is a desired property for compressing long parts of a signal with no significant signal change, numerical precision has to be considered for such cases. In Section 3, we mentioned the rearrangement of Equation (10) for updating the means from [13] to Equation (11), in order to save a multiplication and a division, by slightly sacrificing numerical precision. As we can see, both equations’ right sides can get very small with long segments. Given the sensor values themselves are not next to zero, the right side of Equation (11) possibly gets smaller than the right part of Equation (10) for signals without significant changes, as the new sample and the previous mean are typically very similar values. This results in a small difference divided by a possibly large segment size compared to a sample itself divided by the large segment size. However, for both versions, the numerical precision of the used data types have to be considered for the targeted application and the expected segment length. If longer segments are required, Equation (10) could allow for longer segments depending on the sensors range and expected values, regarding the numerical precision, by trading a bit of processing time. However, in any way, the precision of the data types and threshold value should be chosen according to the desired application.

Another way of avoiding this problem can be achieved by implementing a maximum segment size, which can be chosen according to the available precision of data types. By doing so, a new segment could be produced when either the error value exceeds the threshold or the segment length exceeds the maximum segment size. Note that this variation of the algorithm still does not require a buffer for all sensor samples within a segment.

### 6.2. Computational and Memory Complexities

The computation time of our CPLR algorithm for updating the slope and the error of the current segment for each new data point is independent of the length of the current segment and its computational complexity for each new data point is O(1) with respect to the segment length. As shown in Section 5.3, the processing for each new sample is limited to 203 instructions on an ARM Cortex-M4 microcontroller.

Furthermore, our algorithm has a memory complexity of O(1), since it comes without a buffer. The variables *n*, ${\bar{x^{2}}}_{n-1}$, ${\bar{xy}}_{n-1}$, ${\bar{y^{2}}}_{n-1}$, and ${\hat{\beta }}_{n-1}$ need to be stored globally. Together with TH and the array of segments $\tilde{S}\left[\right]$, which need to store two segmentation points, each consisting of a value and a timestamp (four variables in total), 10 variables have to be stored globally. The algorithm additionally needs 10 local variables for the means ${\bar{x^{2}}}_{n}$, ${\bar{xy}}_{n}$, and ${\bar{y^{2}}}_{n}$, the current slope ${\hat{\beta }}_{n}$, the current segment error $SSR_{n}$, the new sample’s value *y* originating in the previous segment point, the new segments point’s value $\tilde{s}$, and its corresponding timestamp *t*. Note that $\tilde{s}$ and *t* do not necessarily need to be stored in extra variables, but can directly be written to the segments array (line 29). We left them in Algorithm 1 for the sake of readability. Additionally, the new sample value *s*, a pointer to the globally stored segments array $\tilde{S}\left[\right]$, and the index *k* for indicating the position in $\tilde{S}\left[\right]$ for the next segment point are passed as arguments, which makes three additional variables be used. In total, CPLR needs 23 variables. If all integer values are assumed to be 32 bit integers and all floating point numbers are assumed to be single precision floating point types, the data memory consumption is 92 bytes in total, independent of the parameters.

Both aforementioned properties make it possible to implement CPLR on architectures with harsh resource constraints regarding memory and computation time. Moreover, it does not need a maximum buffer size to be specified compared to all other state-of-the-art segmentation algorithms. This reduces the parameter space to be explored for an application specific configuration, i.e., finding an optimal threshold value.

### 6.3. Latency

In real-time applications collecting sensors data online, latency is a crucial property as it directly influences the responsiveness of the system. For online PLA algorithms, this means that a segment approximating a set of sensor samples needs to be output as soon as possible after the corresponding last sensor sample of that segment was sampled. As in emSWAB, only the leftmost segment within the buffer is output, the latency of emSWAB is depending on the buffer size and can result in several sampling periods plus the processing time, which depends on the buffer size as well. For SW, the latency is a single sampling period, as the new sensor sample is decisive for creating a segment up to the previous sample or whether the new sample will be included, plus the processing time of the last sample, which depends on the segment’s length. Our algorithm basically has the same latency behavior as for SW, which is one sample period, but with a constant processing time of the last sample, which involves 203 instructions on an ARM Cortex-M4 microcontroller. Thus, CPLR not only provides a data independent worst-case execution time, but also a small worst-case latency.

## 7. Conclusions

In the paper at hand, we introduced our new CPLR algorithm that supports efficient online piecewise linear approximations of sensor signals with connected segments. In our experiments, we could show that our algorithm can compete with existing state-of-the-art PLA techniques regarding approximation quality. Furthermore, to the best of our knowledge, our CPLR algorithm is the only existing algorithm that provides both a small worst-case execution time with a computational complexity of O(1) and a memory complexity of O(1) leading to the need of only a few variables. These advantages mainly result from the absence of a buffer to store sensor samples.

Finally, due to a short and predictable latency, as well as the deterministic per sample processing times and memory usage, our algorithm satisfies all necessary requirements for performing online piecewise linear approximation on embedded processing units with harsh processing and memory constraints.

## Figures and Tables

**Table 1:** Instruction counts of CPLR, SW, and emSWAB at TH=100 and a buffer size of 100 on an x86-64 architecture.

Algorithm	Min. Instr. Count	Max. Instr. Count	Avg. Instr. Count	Std. Dev.	#Invocations
CPLR	94	150	99.18	15.90	536,146
SW	46	5106	935.20	1115.92	536,146
emSWAB	53	812,807	3423.60	26,628.49	536,175

**Table 2:** Instruction counts of CPLR, SW, and emSWAB on an x86-64 architecture at similar ICRs with a maximum buffer size of 100, evaluated on the ECG data set from Figure 3.

Algorithm	TH	ICR	Min. Instr. Count	Max. Instr. Count	Avg. Instr. Count	Std. Dev.	#Invocations
CPLR	8	0.1943	94	148	104.49	21.36	14,999
SW	10	0.1970	57	1154	306.74	180.26	14,999
emSWAB	8	0.1907	53	181,506	7092.56	17,055.25	15,000

**Table 3:** Instruction counts of CPLR, SW, and emSWAB on an ARM Cortex-M4 microcontroller with a maximum buffer size of 100 for SW and emSWAB.

Algorithm	Minimal Instruction Count	Maximal Instruction Count
CPLR	131	203
SW	59	106+n·56
emSWAB	64	manual analysis infeasible

## Abbreviations

The following abbreviations are used in this manuscript:

WSN	Wireless Sensor Networks
PLA	Piecewise Linear Approximation
SWAB	Sliding Window and Bottom Up
SW	Sliding Window
SSR	Sum of Squared Residuals
OLS	Ordinary Least Squares
ICR	Inverse Compression Ratio
CPLR	Connected Piecewise Linear Regression
ECG	Electrocardiography
MEMS	Micro-Electro-Mechanical Systems
